# Immunovirological response to combined antiretroviral therapy and drug resistance patterns in children: 1- and 2-year outcomes in rural Uganda

**DOI:** 10.1186/1471-2431-11-67

**Published:** 2011-07-26

**Authors:** Laurence Ahoua, Gunar Guenther, Christine Rouzioux, Loretxu Pinoges, Paul Anguzu, Anne-Marie Taburet, Suna Balkan, David M Olson, Charles Olaro, Mar Pujades-Rodríguez

**Affiliations:** 1 Clinical Research Department, Epicentre, Paris, France; 2 Laboratory of Virology, Necker Hospital, Paris, France; 3 Department of Operations, Médecins Sans Frontières, Arua, Uganda; 4 Laboratory of Clinical Pharmacology, Bicêtre Hospital, Kremlin Bicêtre, France; 5 Medical Department, Médecins Sans Frontières, Paris, France; 6 Medical Department, Médecins Sans Frontières, New York, USA; 7 Medical and Administrative Hospital Direction, Arua Regional Referral Hospital, Arua, Uganda

**Keywords:** Children, antiretroviral therapy, Uganda, rural population, patient compliance, drug resistance, pharmacokinetics

## Abstract

**Background:**

Children living with HIV continue to be in urgent need of combined antiretroviral therapy (ART). Strategies to scale up and improve pediatric HIV care in resource-poor regions, especially in sub-Saharan Africa, require further research from these settings. We describe treatment outcomes in children treated in rural Uganda after 1 and 2 years of ART start.

**Methods:**

Cross-sectional assessment of all children treated with ART for 12 (M12) and 24 (M24) months was performed. CD4 counts, HIV RNA levels, antiretroviral resistance patterns, and non-nucleoside reverse transcriptase inhibitor (NNRTI) plasma concentrations were determined. Patient adherence and antiretroviral-related toxicity were assessed.

**Results:**

Cohort probabilities of retention in care were 0.86 at both M12 and M24. At survey, 71 (83%, M12) and 32 (78%, M24) children remained on therapy, and 84% participated in the survey. At ART start, 39 (45%) were female; median age was 5 years. Median initial CD4 percent was 11% [IQR 9-15] in children < 5 years old (n = 12); CD4 count was 151 cells/mm^3 ^[IQR 38-188] in those ≥ 5 years old (n = 26). At M12, median CD4 gains were 11% [IQR 10-14] in patients < 5 years old, and 206 cells/mm^3 ^[IQR 98-348] in ≥ 5 years old. At M24, median CD4 gains were 11% [IQR 5-17] and 132 cells/mm^3 ^[IQR 87-443], respectively. Viral suppression (< 400 copies/mL) was achieved in 59% (M12) and 33% (M24) of children. Antiretroviral resistance was found in 25% (M12) and 62% (M24) of children. Overall, 29% of patients had subtherapeutic NNRTI plasma concentrations.

**Conclusions:**

After one year of therapy, satisfactory survival and immunological responses were observed, but nearly 1 in 4 children developed viral resistance and/or subtherapeutic plasma antiretroviral drug levels. Regular weight-adjustment dosing and strategies to reinforce and maintain ART adherence are essential to maximize duration of first-line therapy in children in resource-limited countries.

## Background

Despite the progress made in the last few years, in 2009 only 356,400 of the estimated 2.5 million children currently infected with HIV were receiving combined antiretroviral therapy (ART) in low- and middle-income countries [1]. Ninety percent of children infected with HIV were living in sub-Saharan Africa and the ART coverage in this area was 26% [2]. In the absence of therapy, more than 50% of HIV-infected children die before the age of 2 years [3]. Therefore, there is an urgent need to increase access to treatment and care and to monitor the effectiveness of therapy.

Comprehensive evaluations of the effectiveness of generic fixed-drug ART in children come mainly from studies conducted in high-income countries [4-8] and, to a lesser extent, from urban areas of resource-poor settings [9,10]. Assessments conducted in rural, resource-limited settings, where programs face important challenges to provide pediatric HIV care, are needed to adapt and improve existing treatment strategies.

In this study, we evaluated pediatric care provision in an HIV/AIDS program in rural, northwestern Uganda. Specifically, we describe survival and program retention, and, among children alive and on ART, patient adherence to therapy and presence of antiretroviral (ARV)-related toxicity. Also presented are plasma non-nucleoside reverse transcriptase inhibitor (NNRTI) concentrations and CD4 and virological responses, including resistance patterns, observed in children with detectable HIV viral load.

## Methods

### Arua Hospital AIDS Care Project

The Arua Hospital AIDS Care Project in Arua, Uganda has provided free treatment and care to patients living with HIV/AIDS since July 2002. Eligibility criteria for ART, patient management, and treatment are based on World Health Organization (WHO) guidelines for scaling up ART in resource-poor settings [11,12].

Pediatric first-line ART doses are adapted to children's weight. At the time of the study, children weighing < 10 kg received pediatric syrups of zidovudine (AZT)-lamivudine (3TC)-nevirapine (NVP); those 10-24 kg, adult fixed-dose combination (FDC) Triviro (3TC-stavudine [d4T]-NVP) or Coviro (3TC-d4T) tablets divided in half; and those ≥ 25 kg, whole tablets of Triviro or Triomune (3TC-d4T-NVP). In case of drug intolerance to NVP, efavirenz (EFV) was used. Due to drug shortages of Coviro and Triviro, between May 2004 and May 2006 children weighting ≥10 kg were given either whole or half Triomune tablets according to body weight. Second-line therapy regimens combined didanosine (ddI)-boosted lopinavir (LPV/r) and either AZT or abacavir (ABC) [13].

Nurses or clinical officers monitored the children every 2 to 6 months to determine clinical stage and diagnosed and treated ARV-related toxicity or intercurrent diseases. CD4 testing was performed at initiation and every 6 months thereafter (every year after 2005). No routine viral load monitoring was performed. Adherence counseling focused on parent/caregiver education. No specialized child psychological support or specific training for pediatric clinical management was offered at that time.

### Study Population

We retrospectively analyzed two observational cohorts of children treated with ART for 12 ± 2 months (M12 cohort) or 24 ± 2 months (M24 cohort). Children alive still followed on treatment were eligible to participate in the cross-sectional survey, conducted between November 2005 and May 2006.

### Study Procedures

We used a standardized questionnaire to collect sociodemographic information, WHO clinical stage data, weight, height, ART information (history of ARV use before enrollment, date of therapy start and regimen, current regimen), reported clinical ARV intolerances (asthenia, lipodystrophy, or gastrointestinal, cutaneous, and neurological symptoms), presence of ARV-related morphological disorders, and adherence to treatment. Two indicators of adherence as reported by parents/caregivers were used: i) percentage of pills taken in the last 4 days (number of pills taken divided by the total number of pills prescribed during the previous 4 days); and ii) percentage of adherence during the last 30 days using a 6-point visual analogue scale (VAS; 0 meaning no medication taken, and 6 all medication taken). For both indicators, poor adherence was defined using a threshold of < 95% [11], moderate adherence as 95-99%, and good adherence as 100%.

Hemoglobin level, platelet count, neutrophil fraction, plasma creatinine level, and transaminase level were measured in the children. Proteinuria and glucosuria was also determined using a urine dipstick technique. Severity of laboratory-based ARV toxicity was graded according to WHO guidelines [14]. CD4 counts were measured using either semi-automated (Cyflow counter, Partec, Münster, Germany) or manual (Dynabead, Dynal Biotech SA, Compiègne, France) techniques. HIV RNA testing was quantified with the automated TaqMan real-time reverse transcription-PCR (RT-PCR) assay (limit of detection 400 copies/mL) [15].

Samples of children with virological failure (viral load ≥ 1,000 copies/mL) were tested for genotypic resistance. Resistance mutation determination was based on the International AIDS Society Resistance Testing-USA panel and resistant virus defined according to the French ANRS resistance algorithm (http://www.hivfrenchresistance.org) [16,17]. High performance liquid chromatography (HPLC) [18,19] was used to determine NVP or EFV plasma concentrations in blood samples collected 12 hours after the last dose intake.

Signed informed consent was sought from all parents or legal caregivers. The study protocol was approved by the Uganda National Council for Science and Technology, the Ugandan AIDS Research Committee, and the Saint-Germain-en-Laye Hospital Consultative Ethics Committee, France.

### Statistical Analysis

Weight-for-height nutritional Z-scores (WHZ) were calculated using WHO growth reference data for children and adolescents [20-22]. Normal NNRTI therapeutic ranges (4,000-8,000 ng/mL for NVP and 1,000-4,000 ng/mL for EFV) [23], were used to define three categories of plasma concentrations: low (below the lower therapeutic limit), normal (within the therapeutic range), and high (above the higher therapeutic limit). Immunological failure was defined according to WHO guidelines [24]: CD4 values < 15% in children of < 36 months, < 10% in the 36-59 month group, and < 100 cells/mm^3 ^in those aged ≥ 5 years.

Children who missed their last appointment for 2 months or more were considered lost-to-follow-up (LFU). Probabilities of survival and care retention were estimated using Kaplan-Meier methods. Patient follow-up was right-censored at the date of the study visit, death, or last visit for children LFU or transferred to another program. All study data were double-entered and analyses performed in Stata 9.0 (Stata Corp., College Station, TX, USA).

## Results

**Figure 1:**
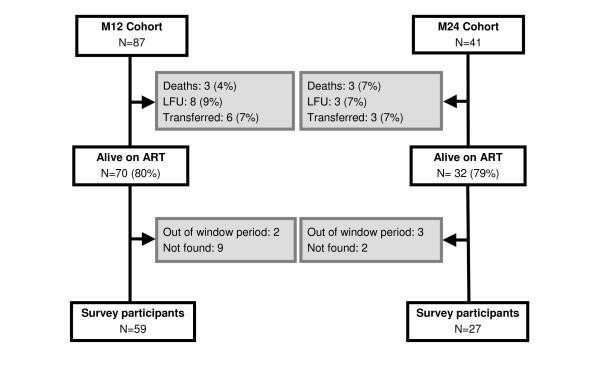
**Cohort profiles, Arua, Uganda, 2006**.

Eighty-seven children had initiated ART 12 ± 2 months (M12 cohort), and 41 children 24 ± 2 months (M24 cohort), before the study inclusion period (Figure 1). Overall, 6 children died, 11 were LFU, and 9 transferred to another program. A total of 102 children (80% of M12 and 79% of M24) were still alive and receiving ART. Of these, 59 (M12) and 27 (M24) children, respectively, participated in the cross-sectional survey (11 patients could not be found and 5 were screened too late).

Of the 6 recorded deaths before the survey, 4 occurred within 6 months of treatment start. Median time to death was 2.3 months [IQR 1.7-7.8]. Probabilities of care retention were 0.91 (95% CI: 0.85-0.95) at 6 months and 0.86 (95% CI: 0.79-0.91) at both 12 and 24 months. Probabilities of survival among children not LFU were 0.97 (95% CI: 0.91-0.99) at 6 months, and 0.95 (95% CI: 0.88-0.98) at both 12 and 24 months.

### Characteristics at ART Initiation

At ART initiation, 39 (45.3%) of the children surveyed were female, median age was 5 years [IQR 3-8], and no children were aged < 12 months (Table 1). Fifty-one (59.3%) children were in clinical stage 3, and 14 (16.3%) in stage 4. Eighteen of 77 (23.4%) children had WHZ < -2. CD4 levels were measured in 38 children at ART initiation. Median CD4 measurement was 11% [IQR 9-15; n = 12] in children aged < 5 years and 151 cells/mm^3 ^[IQR 38-188; n = 26] in children ≥ 5 years.

**Table 1:** Sociodemographic and clinico-immunological characteristics at ART initiation by cohort, Arua, Uganda, 2006

Patient characteristics	M12 cohort		M24 cohort	
	Eligible (N = 70)	Surveyed (N = 59)	Eligible (N = 32)	Surveyed (N = 27)
Demographic factors				
Girls (%)	32 (45)	28 (47.5)	14 (44)	11 (40.7)
Median age, years [IQR]	5.4 [3.2 -8.2]	5.6 [3.2-8.3]	5.5 [4.2-7.7]	5.1 [4.2-7.5]
Age group (%)				
12-35 months	13 (18.6)	12 (20.3)	6 (18.8)	5 (18.5)
36-59 months	19 (27.1)	14 (23.7)	6 (18.8)	6 (22.2)
5-14 years	38 (54.3)	33 (55.9)	20 (62.4)	16 (59.3)

Treatment history				
ARV naïve (%)	67 (95.7)	56 (94.9)	31 (96.9)	26 (96.3)
Median follow-up without ARVs, months [IQR]	5.9 [3.5-11.1]	6.9 [4.2-13.3]	6.3 [1.7-12.0]	6.4 [1.7-12.3]
Clinical factors				
Clinical stage (%)	n = 68	n = 58	n = 32	n = 27
Stage 1/2	16 (23.6)	13 (25.9)	7 (21.9)	5 (18.5)
Stage 3	44 (63.2)	36 (62.1)	18 (56.2)	15 (55.6)
Stage 4	9 (13.2)	7 (12.0)	7 (21.9)	7 (25.9)
Weight-for-height z-score	n = 62	n = 52	n = 29	n = 25
Median [IQR]	-1.1 [-2.0 to -0.5]	-1.2 [-2.1 to -0.6]	-0.8 [-1.6 to -0.1]	-0.8 [-1.8 to -0.3]
< -2 score (%)	17 (27.4)	15 (28.8)	3 (10.3)	3 (12.0)
ART regimen (%)				
AZT 3TC NVP	54 (77.1)	49 (83.1)	25 (78.1)	21 (77.8)
d4T 3TC NVP	16 (22.9)	10 (17.0)	7 (21.9)	6 (22.2)

CD4 testing, median [IQR]				
CD4 cell count ^a^	n = 14	n = 11	n = 17	n = 15
	190 [106-302]	200 [166-302]	70 [22-149]	70 [20-152]
CD4% ^b^	n = 12	n = 10	n = 2	n = 2
	10 [8-14]	11 [8-14]	14 [10-17]	13 [10-17]

**Note**: ART, antiretroviral therapy; 3TC, lamivudine; d4T, stavudine; NVP, nevirapine; AZT, zidovudine.

^a ^Number of children aged ≥ 5 years in M12 = 38 and in M24 = 20.

^b ^Number of children aged < 5 years in M12 = 32 and in M24 = 12.

At therapy start, two children (2.3%) were ART-experienced (M12 cohort). Two others had received prevention of mother-to-child HIV infection prophylaxis, one patient received short-course AZT together with single-dose NVP (M12 cohort), and one patient received single-dose NVP (M24 cohort). Seventy (81.4%) children were started on AZT-3TC-NVP. A greater proportion of children had received ARV pediatric formulation in the M12 than in the M24 cohort (72.9% vs. 14.8%).

### Characteristics at Survey

Mothers were the main caregivers for 50.8% of children in the M12 cohort but only for 37.0% in the M24 cohort (Table 2). Twenty-one percent of all children were orphans.

**Table 2:** Patient characteristics at survey evaluation by cohort, Arua, Uganda, 2006

Children aged < 15 years	M12 cohort (N = 59)	M24 cohort (N = 27)
Type of caregiver (%)		
Mother	30 (50.8)	10 (37.0)
Father	6 (10.2)	3 (11.1)
Another relative	21 (35.6)	13 (48.2)
Other	2 (3.4)	1 (3.7)
Father receiving ART (%)	17 (28.8)	4 (14.8)
Mother receiving ART (%)	29 (49.2)	11 (40.7)
Both parents died (%)	12 (20.3)	6 (22.2)

Clinico-immunological characteristics		
Cumulative clinical stage (%)		
Stage 1/2	13 (22.0)	3 (11.1)
Stage 3	26 (44.1)	10 (37.0)
Stage 4	20 (33.9)	14 (51.9)
Weight gain, kg, median [IQR]	4 [3-5]	5 [4-7]
Weight-for-height z-score (%)	n = 51	n = 22
< -2 z-score	2 (3.9)	2 (9.1)

Reported ARV-related toxicity		
Asthenia	8 (13.6)	2 (7.4)
Gastrointestinal symptoms	25 (42.4)	12 (44.4)
Neurological disorders	23 (39.7)	10 (37.0)
Morphological disorders	13 (22.0)	5 (18.5)

Adherence to ART		
4-day recall (%)		
Good (100%)	49 (83)	23 (85)
Moderate (95-99%)	10 (17)	4 (15)
Poor (< 95%)	0 (0)	0 (0)
30-day VAS^a ^(%)		
Good (100%)	28 (48)	19 (70)
Moderate (95-99%)	22 (38)	5 (19)
Poor (< 95%)	8 (14)	3 (11)

Median CD4 testing [IQR]		
CD4 cell count, cell/μL	n = 33	n = 16
	504 [399-715]	210 [124-458]
CD4%	n = 26	n = 11
	25 [21-29]	20 [16-27]

Plasma EFV concentrations (%)	n = 5	
Low (< 1,000 ng/mL)	1 (20.0)	-
High (> 4,000 ng/mL)	1 (20.0)	
Plasma NVP concentrations (%)	n = 54	n = 27
Low (< 4,000 ng/mL)	18 (33.3)	6 (22.2)
High (> 8,000 ng/mL)	9 (16.7)	2 (7.4)

**Note**: ARV, antiretroviral; ART, antiretroviral therapy; EFV, efavirenz; IQR, interquartile range; NVP, nevirapine; VAS, visual analogue scale.

^a^Missing information for one patient in the M12 cohort.

Using the 4-day recall adherence indicator, all children were classified as fully or moderately adherent to ART (Table 2). The 30-day VAS classified 14% (M12) and 11% (M24) of children, respectively, as poorly adherent to ART (< 95% score).

Gastrointestinal symptoms (43.0%) and peripheral neuropathy (38.4%) were the most frequently reported ARV-related toxicities. Morphological disorders were diagnosed in 20.9% (18/86) of children, including abdominal adipose tissue increase (15.1%, n = 13), lower limb muscle loss (4.7%, n = 4), gluteal muscle loss (3.5%, n = 3), breast adipose tissue increase (1.2%, n = 1), and facial muscle loss (1.2%, n = 1). Grade 1 or 2 neutropenia was the laboratory-related toxicity most frequently observed (16.3%), and severe toxicity was found in only one child (neutropenia of grade 3, M12).

Three children (11.1%) were in immunological failure at the time of the study; all were aged ≥ 5 years and received treatment for 2 years. In the M12 cohort, median CD4 percent gain was 11% [IQR 10-14; n = 10] in children aged < 5 years, and CD4 count gain 206 cells/mm^3 ^[IQR 98-348; n = 11] in those of ≥ 5 years. In the M24 cohort, the CD4 percent gain was 11% [IQR 5-17; n = 2] in children aged < 5 years, and CD4 count gain 132 cells/mm^3 ^[IQR 87-443; n = 15] in the elder group.

### Plasma NNRTI Concentrations

**Figure 2:**
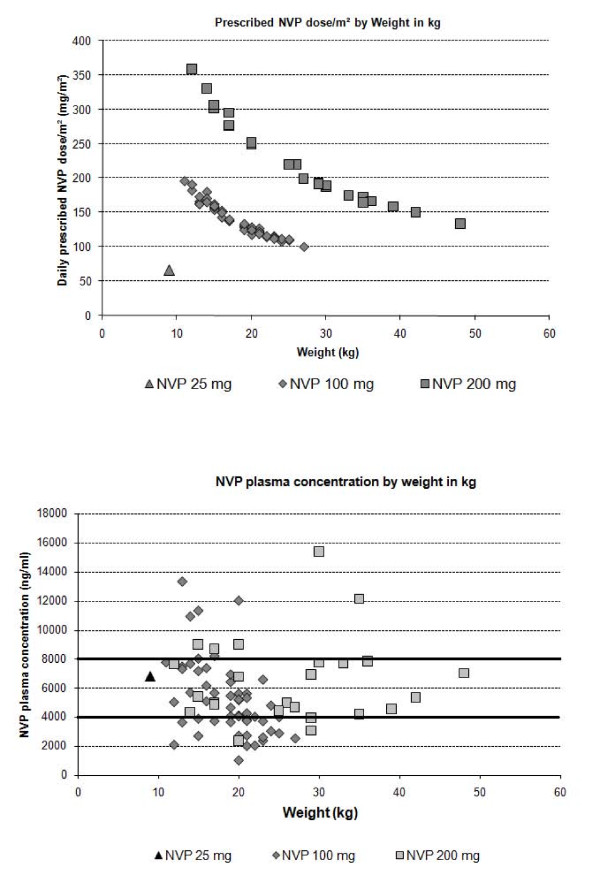
**Association between weight and (2a) daily NVP, and (2b) NVP plasma concentration, Arua, Uganda, 2006**. 2a) Prescribed daily NVP per body surface area (mg/m^2^) by children's weight (kg) 2b) NVP plasma concentration (ng/mL) by children's weight (kg).

Plasma concentrations of NNRTIs were measured in all children: 81 receiving NVP-based and 5 EFV-based therapy. Median prescribed NVP dose per body surface area was 150.3 mg/m^2 ^[IQR 124.1-184.9] and decreased with increasing weight (Figure 2a). Subtherapeutic NNRTI plasma levels were detected in 32.2% (19/59) and 22.2% (6/27) of children at M12 and M24, respectively (Table 2). Only children weighing < 30 kg were found to be underdosed for NVP (37.0% of those who were prescribed 100 mg, 18.8% of those on 200 mg, and none of those on 25 mg of NVP twice daily; Figure 2b). Those weighing 20-30 kg were more frequently underdosed for NVP than other children (51.9% compared to 21.3% for children of < 10 kg; *P *= 0.005). Furthermore, 16.9% (10/59) of M12 and 7.4% (2/27) of M24 children had high plasma concentrations of NNRTIs (NVP or EFV).

### Virological Response and Drug Resistance

HIV RNA suppression (< 400 copies/mL) was observed in 59.3% (35/59) of M12 and 33.3% (9/27) of M24 children (Table 3). Treatment adherence was not associated with either viral suppression (overall Fisher's exact *P *= 0.42 for VAS and 1.00 for 4-day recall) or presence of low NNRTI plasma concentrations (*P *= 0.35). Similarly, no association was observed between weight and viral suppression (*P *= 0.17), and, among children with detectable HIV RNA, weight was not correlated with viral load measurements (overall Spearman's correlation coefficient 0.07, and 0.05 for M12 only).

**Table 3:** Results of virological and genotypic resistance testing by cohort, Arua, Uganda, 2006

	M12 cohort (N = 59)	M24 cohort (N = 27)
HIV RNA < 400 copies/mL (%; 95% CI)	35 (59.3; 46.1-71.3)	9 (33.3; 17.5-54.0)
HIV RNA 400-999 copies/mL (%; 95% CI)	7 (11.9; 5.6-23.3)	1 (3.7; 0.5-24.5)
HIV RNA ≥ 1,000 copies/mL (%; 95% CI)	17 (28.8; 18.5-42.0)	17 (62.9; 42.5-79.7)
Available genotypic resistance (%)	17 (100)	17 (100)
Wild type virus (%)	3 (17.6)	1 (5.9)
Resistance to ≥ 1 ARV drug (%)	13 (76.5)	16 (94.1)
Resistance to EFV and NVP (%)	0 (0)	0 (0)
Resistance to 3TC, EFV and NVP (%)	12 (70.6)	6 (35.3)
Extensive resistance^a ^(%)	0 (0)	8 (47.1)

**Note**: ARV, antiretroviral; 95% CI, binomial 95% confidence interval; EFV, efavirenz; NVP, nevirapine; 3TC, lamivudine.

**^a^**Resistance to EFV, NVP, 3TC, stavudine, zidovudine or tenofovir and didanosine.

Genotypic resistance testing was successful in all 34 specimens with viral load ≥ 1,000 copies/mL (Table 3). HIV-1 subtypes were A1 (n = 7), C (n = 1), and D (n = 4), and for 22 children the subtype was not specified. Wild-type viruses were found in 4 patients (3 at M12 and 1 at M24). The most prevalent mutations were M184V (nucleoside reverse transcriptase inhibitors [NRTIs]) and Y181C (NNRTIs). Twenty-nine (85.3%) children had at least 1 major mutation conferring drug resistance to both NRTIs and NNRTIs. Prevalence of detectable resistant virus obtained after excluding the 8 children who had 400-999 HIV RNA copies/mL was 25% (13/52) at M12 and 62% (16/26) at M24. Twelve (M12) and 6 (M24) children had viruses resistant to both 3TC and NNRTIs (EFV and NVP). Eight specimens from the M24 cohort had K65R, T69A, V751, K103N, F116Y, Q151M, V179S, Y181C, and M184V mutations, conferring resistance to all NRTIs and to EFV and NVP.

## Discussion

In these two pediatric cohorts of children followed in rural Uganda, we found retention rates of 86% and sustained immunological responses 1 and 2 years after ART start. Nevertheless, virological responses were suboptimal, as only 59% of M12 and 33% of M24 children achieved HIV viral suppression, and resistant viral strains were found in 25% and 62% of children, respectively.

As in other sub-Saharan African cohorts [25-28], all children followed in our program were enrolled and started therapy after the first year of life, median age at inclusion being 5 years old. Given that young, untreated children are at high risk of rapid disease progression and death before the age of 2 years [3,29-32], the overall impact of HIV/AIDS programs in children living in these settings is likely to be suboptimal. Thus, delay in diagnosis of pediatric HIV infection and lack of availability of integrated HIV care and treatment in maternal and child health services at the time of the survey were likely to be partly responsible for the late start of therapy in our site.

Furthermore, as in other pediatric cohorts in resource-limited settings [26,27,33,34], children in our program started therapy at advanced clinico-immunological stage and the majority of deaths and LFU occurred early in treatment. Delayed diagnosis of HIV infection and failure to diagnose and treat concurrent life-threatening infections such as tuberculosis, pneumonia, or sepsis [35] might be responsible for the early losses reported. Starting ART before 12 weeks of life has recently been associated with a 76% reduction in child mortality compared to deferred therapy until immunological or clinical progression is diagnosed [36]. Therefore, some of the early losses in our cohort could have been prevented if ART was started earlier. Despite this, we observed overall high and sustained survival and retention rates in children treated with ART up to 2 years after therapy start. These findings are consistent with reports from other programs from resource-limited settings [10,25,37].

Although direct comparison of immunological responses across studies is not possible because of its high dependence on patient age [38], we observed significant CD4 T-cell responses to ART independent of the virological status of the patient, and, as previously reported [27,28,38,39], greater increases were seen in the first than in the second year of therapy among children aged ≥5 years (gains of 206 vs. 132 cells/mm^3^).

The small sample size in our survey must be considered when interpreting these findings. The small numbers of patients did not allow investigating risk factors for virological failure. Estimates reported in other studies range from 49% in Abidjan, Côte d'Ivoire, to 80% in 63 children exposed to single-dose NVP in Durban, South Africa, at one year of therapy [9,27,40-42]. In the urban studies in Côte d'Ivoire and South Africa, 49% virological suppression was reported at 2 years of ART [9,27]. Large differences in virological response rates have been observed between clinical studies among children, and the majority showed lower rates than in adults [38]. Possible explanations for such discrepancies could be differences in patient characteristics at ART initiation, regimens used, pediatric dose formulations, drug pharmacokinetics, and/or inadequate compliance to treatment.

As mentioned before, most children in our cohort started treatment at an advanced stage of disease. Presence of high HIV RNA at therapy start has been associated with slower decay rates of plasma viral levels and longer time to reach undetectable viral levels [42-45]. Increased risk of virological failure has also been reported in children with CD4 < 5% [40]. Our patients received NNRTI-based therapy, and most were given NVP. Higher rates of virological success have been shown in young patients treated with protease inhibitors than in those using NNRTI regimens in some [9] but not all studies [27,46]. Furthermore, virological response has also been shown to vary in clinical studies using the same medication [38].

Ensuring adequate administration of ART to children is difficult and requires continuous dose adjustment in response to rapid changes in height and weight related to growth. In our cohorts, 1 in 4 children had subtherapeutic NVP plasma concentrations, especially children in the 20-30 kg weight range, highlighting the need for close monitoring of treatment, clear and simplified treatment guidelines, and availability of appropriate pediatric dose formulations.

Ensuring and maintaining good treatment adherence in children is also challenging, especially in resource-limited settings, since many children are orphans or live in difficult social situations: 1 in 5 patients were orphans in our study, and 43% had a caregiver other than a parent. Furthermore, palatable syrups or appropriate pediatric tablet formulations are expensive and not broadly available. In our study, 13% of children were identified as poorly adherent to ART, but this is likely to be underestimated since it was based on parental or caregiver reports. Four children with virological failure had wild-type virus and had therefore not been taking any treatment for some time. Furthermore, the prevalence of resistance mutations was relatively high but similar to those reported by a previous study in Côte d'Ivoire [26]. A recent review of studies investigating ART adherence in children treated in low- and middle-income countries identified difficult familial situations and low socioeconomic status, absence of parental and/or child HIV status disclosure, complicated regimens, and drug-related adverse events as barriers to treatment adherence [47,48]. Age-adapted therapeutic education of children and extensive discussions and support to parents/caregivers need to be encouraged to improve ART uptake in this vulnerable group.

As reported by others [38,49], we did not observe severe adverse drug reactions at the time of the survey, apart from one patient with severe neutropenia. However, 3 of 7 children reported gastrointestinal symptoms, which might interfere with drug absorption and affect treatment adherence [50]. Peripheral neuropathy and morphological disorders were not uncommon (38% and 21% of children, respectively). One longitudinal and one cross-sectional study also reported relatively high rates of lipodystrophy in children treated with d4T or protease inhibitors (29% and 33% of children, respectively), especially in those who started therapy at an advanced stage of disease [51,52]. This syndrome is frequently associated with metabolic abnormalities such as hyperinsulinemia and dyslipidemia. However, its causal link to ART is to be established, and its long-term consequences for the treatment of children are unknown [52].

## Conclusions

Important challenges need to be tackled to improve HIV pediatric care in resource-limited settings. These include increasing early HIV diagnosis through programs for the prevention of mother-to-child HIV transmission, training in pediatric clinical management and adherence counseling, and development of palatable and simplified pediatric drug formulations.

## Abbreviations

ABC: abacabir; ART: combined antiretroviral therapy; ARV: antiretroviral; AZT: zidovudine; d4T: stavudine; ddI: didanosine; EFV: efavirenz; FDC: fixed-dose combination; HPLC: high performance liquid chromatography; LFU: lost to follow-up; LPV/r: boosted lopinavir with ritonavir; NNRTI: non-nucleoside reverse transcriptase inhibitor; NRTI: nucleoside reverse transcriptase inhibitor; NVP: nevirapine; VAS: visual analogue scale; WHO: the World Health Organization; WHZ: weight-for-height nutritional Z-scores.

## Competing interests

The authors declare that they have no competing interests.

## Authors' contributions

LA designed and implemented the study, analyzed and managed data, interpreted results, and wrote the manuscript. GG, PA, and CO set up and implemented the study in the field and contributed to the interpretations of results. LP prepared the study's EpiData database and performed data management. CR and A-MT performed the virological and genotypic testing and contributed to interpretation of these results. SB and DMO helped to design the study, interpret the results, and draft the manuscript. MP-R managed the data, performed statistical analysis, interpreted results, and co-wrote the manuscript. All authors read and approved the final manuscript.

## Pre-publication history

The pre-publication history for this paper can be accessed here:

http://www.biomedcentral.com/1471-2431/11/67/prepub
